# HMOX1 STR polymorphism and malaria: an analysis of a large clinical dataset

**DOI:** 10.1186/s12936-022-04352-x

**Published:** 2022-11-17

**Authors:** Fergus Hamilton, Ruth Mitchell, Aubrey Cunnington, Peter Ghazal, Nicholas J. Timpson

**Affiliations:** 1 MRC Integrative Epidemiology Unit, University of Bristol, Oakfield Grove, Bristol, BS8 2BN UK; 2 Infection Sciences, North Bristol NHS Trust, Bristol, England UK; 3 Department of Infectious Disease, Faculty of Medicine, Imperial College London, London, UK; 4 Centre for Paediatrics and Child Health, Imperial College London, London, UK; 5 System Immunity Research Institute, Division of Infection and Immunity, Cardiff University, Cardiff, UK

## Abstract

**Background:**

Inducible expression of heme oxygenase-1 (encoded by the gene *HMOX1*) may determine protection from heme released during malaria infections. A variable length, short tandem GT(n) repeat (STR) in *HMOX1* that may influence gene expression has been associated with outcomes of human malaria in some studies. In this study, an analysis of the association between variation at the STR in *HMOX1* on severe malaria and severe malaria subtypes is presented in a large, prospectively collected dataset (MalariaGEN).

**Methods:**

The HMOX1 STR was imputed using a recently developed reference haplotype panel designed for STRs. The STR was classified by total length and split into three alleles based on an observed trimodal distribution of repeat lengths. Logistic regression was used to assess the association between this repeat on cases of severe malaria and severe malaria subtypes (cerebral malaria and severe malarial anaemia). Individual analyses were performed for each MalariaGEN collection site and combined for meta-analysis. One site (Kenya), had detailed clinical metadata, allowing the assessment of the effect of the STR on clinical variables (e.g. parasite count, platelet count) and regression analyses were performed to investigate whether the STR interacted with any clinical variables.

**Results:**

Data from 17,960 participants across 11 collection sites were analysed. In logistic regression, there was no strong evidence of association between STR length and severe malaria (Odds Ratio, OR: 0.96, 95% confidence intervals 0.91–1.02 per ten GT(n) repeats), although there did appear to be an association at some sites (e.g., Kenya, OR 0.90, 95% CI 0.82–0.99). There was no evidence of an interaction with any clinical variables.

**Conclusions:**

Meta-analysis suggested that increasing HMOX1 STR length is unlikely to be reliably associated with severe malaria. It cannot be ruled out that repeat length may alter risk in specific populations, although whether this is due to chance variation, or true variation due to underlying biology (e.g., gene vs environment interaction) remains unanswered.

**Supplementary Information:**

The online version contains supplementary material available at 10.1186/s12936-022-04352-x.

## Background

The gene *HMOX1* (also known as heme-oxygenase 1) encodes for a protein HO-1 that has been shown to be critical for survival in cerebral malaria in mouse models of malaria [1, 2]. HO-1 breaks down heme (or haem) to iron, biliverdin and carbon monoxide, with the latter playing a protective role in experimental cerebral malaria [1]. In a non-coding region of the gene, there exists a short tandem repeat (STR), that shows a GT(n) polymorphism of variable length. Some but not all *in-vitro* studies have suggested that the length of this STR associates with expression of *HMOX1*, where cells containing longer STRs (more than around 35 repeats) have reduced inducible expression [2]. The STR ranges from around 25 repeats to around 40, with wide ranging differences across different populations [3].

Multiple human studies across many diseases have suggested that STR variation at this locus is associated with relevant clinical outcomes. For example, a meta-analysis in diabetes suggested an effect of this STR (longer repeats increasing the risk of type II diabetes) [4], while another meta-analysis identified longer repeats decreasing the risk of neonatal jaundice [5]. Additionally, on the basis of pathway driven basic science and given the association with mammalian malaria, multiple studies in malaria have examined the role of this STR in both the incidence of malaria and development of severe malaria (e.g., cerebral malaria) [1, 6, 7]. Despite this, evidence of an effect of STR variation on the presence of severe malaria remains inconclusive, with studies having small sample sizes ( < 1000 participants) and variable definitions of STR length. Furthermore, given the nature of STR variation and recent evolution, controlling for ethnicity in both case status and STR length is key to understanding if there is a relationship between the *HMOX1* STR and malaria outcomes, which requires some adjustment for population structure which has not been performed in studies so far.

This study aimed to impute the *HMOX1* STR using a recognized haplotype reference panel for STRs [8] in a large, geographically diverse case–control study of malaria (MalariaGEN [9]) in order to analyse the association between STR genotype and (a) malaria case status (b) severe malaria subtype status.

## Methods

### Reporting guidelines

This study was reported in line with the STREGA reporting guidelines (ref https://journals.plos.org/plosmedicine/article?id=10.1371/journal.pmed.1000022) which are available in supplement S1

### Data source

This analysis used the MalariaGEN dataset and was approved by the MalariaGEN IDAC (Application: 71). Clinical phenotyping (clinical parameters, parasite count) was available for one subset of this cohort, the Kenya cohort, and was arranged via Professor Tom Williams at the KEMRI-Wellcome Unit [10].

This study was a meta-analysis of 11 separate studies on resistance to severe malaria. Each study contributed to a wider meta-analysis of resistance to severe malaria [9]. Each site had the same genetic analysis pipeline (detailed in the above publication), with analysis performed at each site, and then meta-analysed for the summary results.

### HMOX1 STR genotyping and definition of STR length

This analyses used the MalariaGEN data set. MalariaGEN SNP array data was downloaded from the EGA (EGA, dataset EGAD00010001799) [9]. Imputation was performed using a recently developed haplotype reference panel based on the 1000 Genomes data using Beagle v4.2 [8] Previous work has shown this imputation is reliable and have shown no benefit of filtering this imputation based on predicted genotype probability [11]. In that study, data from the 1000 Genomes Project [12] and the Human Genome Diversity project [13] was used to compare the imputed STR length with the real STR length, as called by whole genome sequencing. In general, correlation between real and imputed STR length in worldwide populations was good (Pearson’s R > 0.8), although there was worse imputation performance in some ethnic backgrounds. Previous work provides detail on the imputation approach and quality control [11].

Previous literature on *HMOX1* STR length in malaria has used a wide range of reference lengths for the *HMOX1* STR [14]. Given the lack of consensus and evidence for any given definition the decision was made to use the summed repeat length as our primary definition in this study, where STR length is defined by the total number of GT repeats. For further analysis, the repeat was split into three alleles—short (S), medium (M), and long (L), using definitions below, in line with previous literature and the trimodal variation at this STR. Genotypes were subsequently defined by the combination of these alleles, e.g. SS, SM, etc., in line with previous literature, leading to six genotypes at this locus [3].

#### Malaria outcomes

Malaria outcomes were extracted from the MalariaGEN dataset provided by MalariaGEN alongside data on sex, ethnicity, and country. Outcomes included: case status and type of severe malaria status (cerebral malaria (CM) and/or severe malarial anaemia (SMA). Details of definitions are with the original MalariaGEN publication [9].

For one MalariaGEN collection site (Kenya), more detailed clinical phenotyping of malaria cases was available (blood pressure, platelet count, haemoglobin, MCV, parasite count, severe renal disease, and mortality). Details of definitions are with the relevant publication [10].

### Statistical approach

#### Main outcome

The primary analytic method was logistic regression on severe malaria case status in each individual study site with the total STR repeat length as the explanatory variable. Subsequently, site-specific estimates were meta-analysed in a random effects model to generate summary estimates across the whole cohort. For each severe malaria subtype (CM, SMA, or both), a similar analysis was performed comparing cases of each severe malaria subtype vs a) all other cases, and b) vs all controls. Subsequently, an alternative repeat length definition was generated by splitting each allele across the trimodal distribution into short (S), medium (M), long (L) alleles. The allele cut points were short ( < 27 repeats), medium (27–32 repeats) and long  > 32 repeats and these alleles were applied in a logistic regression against severe malaria case status.

As a third alternative model, the actual genotype was included (e.g., short-short, short-medium, medium-long, long-long), in a logistic regression model against the reference genotype (medium-medium). These analyses were again performed for each outcome in each country and meta-analysed in a random effects model. To ensure estimates had some precision, models were only run if they included more than 30 cases, and more than 150 total patients.

The first 10 principal components and sex were included as covariates in our models. These principal components were supplied by MalariaGEN and calculated at the site level, and therefore represent genetic variation at each site, not across the whole meta-analysis.

#### Associations and interactions with clinical variables

One data set had available clinical data (Kenya). At this site, linear regression was performed with STR length on the clinical biomarkers of severity: Hb, platelet count, blood pressure, white cell count, MCV, and platelet count, and parasite count (logged to improve model fit).

Subsequently, an assessment was made of any potential intraction between STR length and clinical variables. In these models, logistic regression was performed on severe malaria case status with an interaction fitted between repeat length and clinical variable (e.g. case ~ STR length * platelet count). This analysis was based on laboratory data suggesting that *HMOX1* variation may only have clinical impact in certain subtypes (e.g. high parasite counts) of severe malaria.

## Results

SNP array and clinical data were available for 17,960 participants in MalariaGEN. All genotypes were successfully imputed. 215 parents and 3 patients had unknown case status, and 302 patients who had no country information. These were excluded from the analysis data set leading to 8658 cases, and 8931 controls. Table 1 shows the breakdown of case status and severe disease by MalariaGEN site.

**Table 1:** Distribution of severe malaria across the MalariaGEN cohort

Country	Total number	Cases (% total)	SMA (% cases)	CM (% cases)	Both (% cases)
Burkina Faso	1327	733 (55.2%)	28 (3.8%)	94 (12.8%)	18 (2.5%)
Cameroon	1277	592 (46.4%)	66 (11.1%)	32 (5.4%)	8 (1.4%)
Gambia	5091	2487 (48.9%)	456 (18.3%)	780 (31.4%)	134 (5.4%)
Ghana	716	396 (55.3%)	41 (10.4%)	31 (7.8%)	5 (1.3%)
Kenya	3261	1646 (50.5%)	174 (10.6%)	690 (41.9%)	189 (11.5%)
Malawi	2499	1182 (47.3%)	65 (5.5%)	642 (54.3%)	109 (9.2%)
Mali	446	263 (59%)	81 (30.8%)	61 (23.2%)	51 (19.4%)
Nigeria	131	109 (83.2%)	1 (0.9%)	28 (25.7%)	0 (0%)
PNG	770	396 (51.4%)	115 (29%)	49 (12.4%)	7 (1.8%)
Tanzania	807	409 (50.7%)	178 (43.5%)	31 (7.6%)	25 (6.1%)
Vietnam	1264	718 (56.8%)	23 (3.2%)	154 (21.4%)	4 (0.6%)

Case frequency differed by country with the highest number of cases in Nigeria (83% of included participants cases), and the lowest in Cameroon (46% of included participants cases). The presence of severe malaria subtypes also differed greatly by site, with rates of severe malarial anaemia as high as 31% in Mali, and as low as 1% in Nigeria.

Ethnicity had an equally large impact on case ratio and severe malaria subtypes, with Additional file 1: Table S1 summarizing this data.

Details of the *HMOX1* STR imputation process and accuracy are detailed in a recent publication on the HMOX1 STR in UK Biobank [11].

**Fig. 1:**
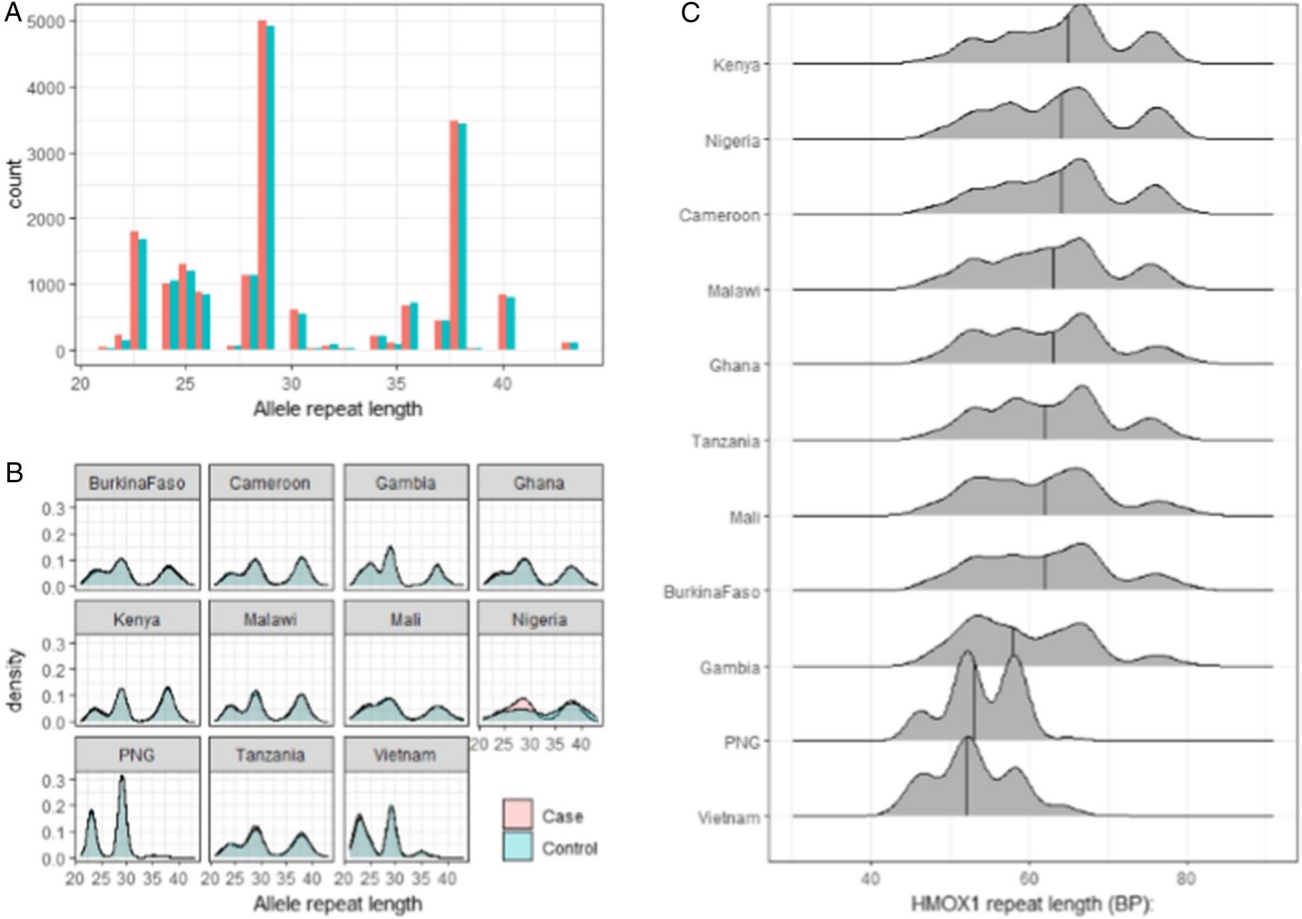
**A** STR length of each allele across MalariaGEN, **B** STR length across each site, and **C** summed STR length across each site (line represents median STR length)

As expected, *HMOX1* STR genotype differed across populations. Figure 1A shows the distribution of STR lengths across the whole of MalariaGEN, while Fig. 1B shows individual distributions of alleles stratified by case/control status and Fig. 1C shows the summed STR length across both alleles for all countries. The trimodal distribution (peaks at 29 STRs, 38 STRs, and 23 STRs) previously reported was identified, although all three peaks were not present in all subpopulations. The mean total repeat length across all cohorts was 60.9 repeats, with a standard deviation of 8.2 repeats.

### Primary analysis

**Fig. 2:**
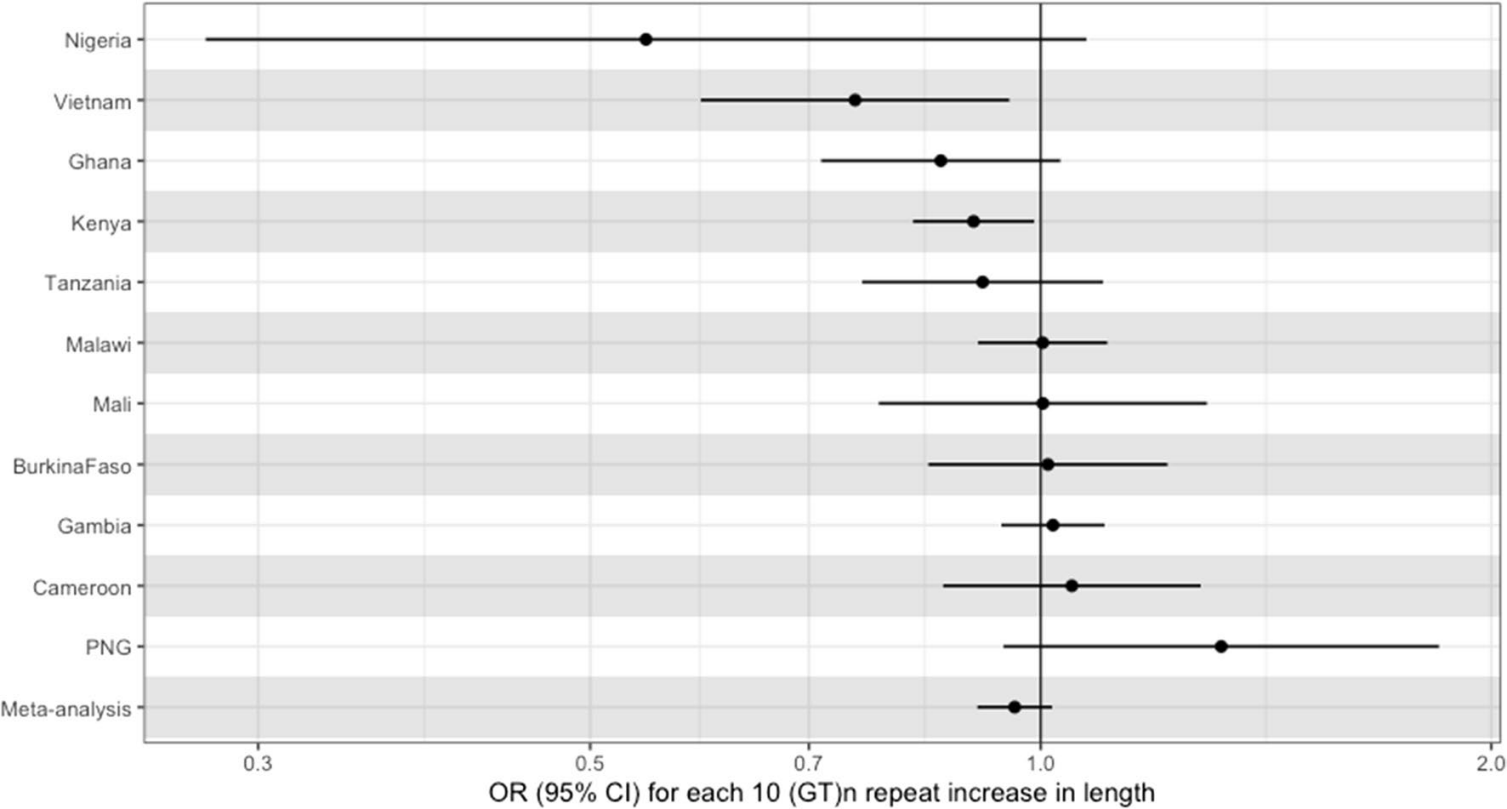
Effect of HMOX1 repeat length on severe malaria case status across each site

The primary analysis consisted of logistic regression of severe malaria cases status on *HMOX1* repeat length, performed at each site individually, then meta-analysed the results using random-effects meta-analysis. Overall, the meta-analysed effect estimate confidence intervals spanned the null (0R 0.96, 95% CI 0.91–1.02), but was consistent with a small negative effect (e.g., increasing repeat length being protective against severe malaria). There was evidence of variation in effect estimates across the sites (p value for heterogeneity = 0.06), and of variation in effects size across populations. For example, increasing STR length was associated with decreased odds of severe malaria in Nigeria (OR 0.54 for a 10 GT(n) repeat increase in repeat length, 95% CI 0.28–1.07, p = 0.07), but higher odds of severe malaria in Papua New Guinea (OR 1.32; 95% CI 0.94–1.85, p = 0.10). Full results are shown in Fig. 2 and Table 2.

**Table 2:** Effect estimates for each country for our primary analysis

Country	Estimate (95% CI)	p value
PNG	1.32 (0.94–1.85)	0.104
Cameroon	1.05 (0.86–1.28)	0.635
Gambia	1.02 (0.94–1.1)	0.641
Burkina Faso	1.01 (0.84–1.22)	0.906
Malawi	1 (0.91–1.11)	0.953
Mali	1 (0.78–1.29)	0.979
Tanzania	0.91 (0.76–1.1)	0.344
Kenya	0.9 (0.82–0.99)	0.029
Ghana	0.86 (0.71–1.03)	0.101
Vietnam	0.75 (0.59–0.95)	0.018
Nigeria	0.54 (0.28–1.07)	0.079
Meta-analysis	0.96 (0.91–1.02)	0.171

**Fig. 3:**
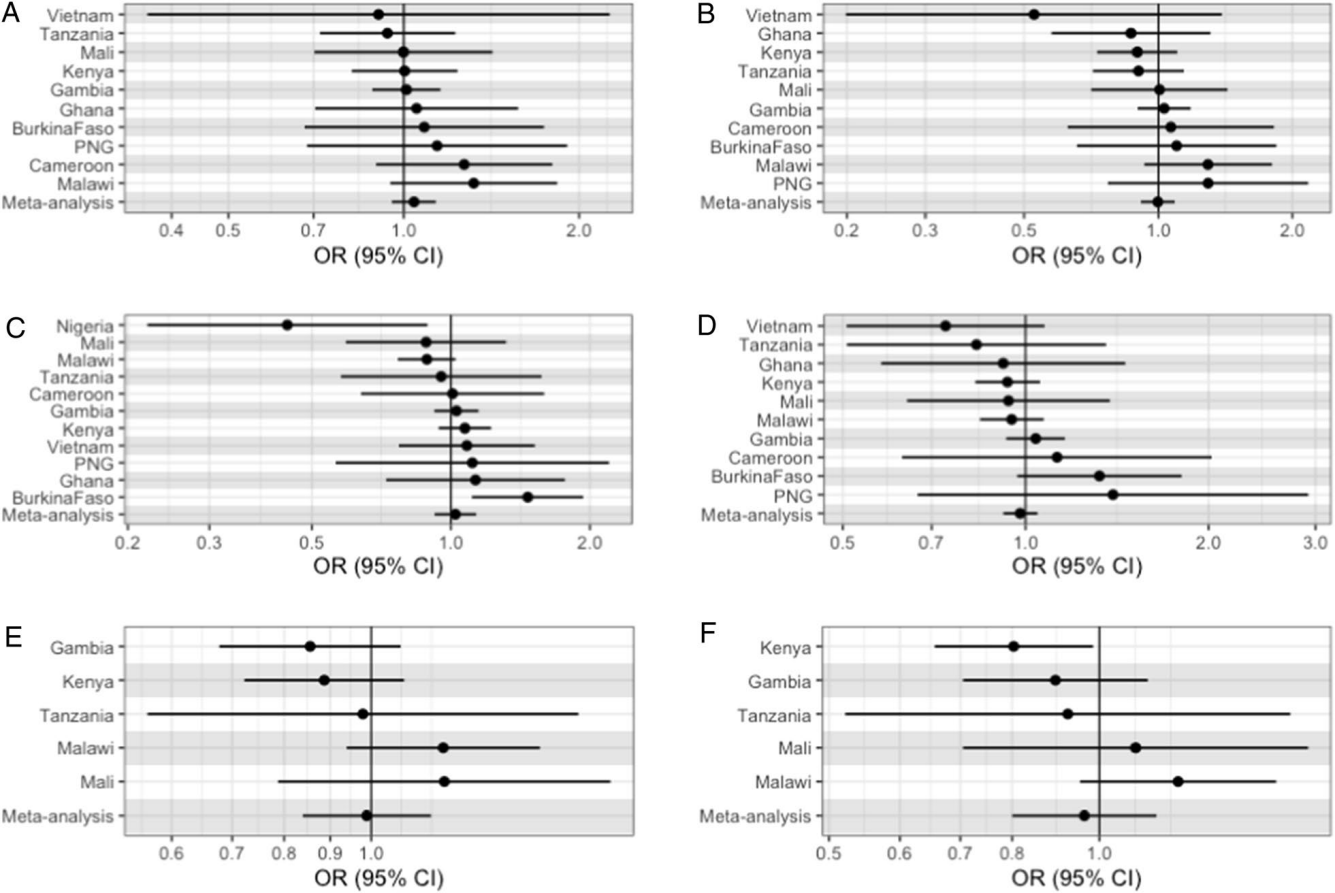
Effect estimates of the effect of HMOX1 repeat length in severe malaria subtypes: **A** CM, vs all other cases, **B** CM, vs all controls, **C** SMA vs all other cases, **D** SMA vs all other controls, **E** Both vs all other cases, **F** Both vs all controls

For the severe malaria subtypes (CM, SMA, and these combined), there was no strong evidence of a consistent effect across sites with any severe malaria subtype, with all estimates confidence intervals crossing the null (Fig. 3A–F and Additional file 1: Table S2).

### Allelic and genotypic model

Allelic and genotypic models were generated by classifying the repeat length into three alleles (S, M, L), and 6 genotypes (SS, SM, MM, ML, LS, LL). Table 3 describes the number of included participants with each genotype.

**Table 3:** Genotypes of included participants

MM	LL	MS	SS	LS	LM
2708	2164	3880	1625	2938	4274

There was weak evidence of an association between *HMOX1* STR genotype and the outcome “any severe malaria subtype”. The strongest evidence for association was seen for the primary outcome of severe malaria and SM genotype, which reported an OR of 1.15 (95% CI 1.01–1.13) (Additional file 1: Figure S2). All genotypes were associated with an OR of  > 1 (Additional file 1: Table S3), suggesting the reference genotype had the lowest risk of severe malaria, although effect estimates were imprecise and confidence intervals cross the null. In contrast, our allelic model showed no clear association between the number of L, M, or S alleles carried and the development of severe malaria, with wide uncertainty around effect estimates (Additional file 1: Figure S3).

#### Interaction with clinical variables

Detailed clinical metadata was available for one clinical site (Kenya) and was used to assess any association between the *HMOX1* STR and clinical variables. Demographics of the included cohort, split by case status, are available in Table 4.

**Table 4:** Characteristics of the Kenya cohort [10]

Characteristic	Both, N = 182	Cerebral Malaria, N = 657	Other^2^, N = 387	Severe Malarial anaemia, N = 169
Mortality	34 (19%)	100 (15%)	29 (7.5%)	15 (8.9%)
Renal Disease	97 (53%)	218 (33%)	139 (36%)	16 (9.5%)
Diastolic BP	49 (42, 59)	58 (49, 66)	55 (48, 64)	50 (45, 58)
Unknown	84	231	147	67
Systolic BP	94 (84, 105)	99 (90, 110)	96 (86, 104)	93 (84, 102)
Unknown	82	207	138	65
Haemoglobin	4.00 (3.20, 4.50)	7.70 (6.20, 9.30)	6.90 (5.50, 8.58)	3.80 (3.20, 4.50)
Unknown	0	0	1	0
Platelet count	101 (63, 159)	117 (65, 250)	107 (64, 182)	106 (68, 154)
Unknown	17	27	14	6
MCV	73 (68, 80)	74 (67, 79)	73 (67, 79)	74 (68, 81)
Unknown	1	0	1	0
WBC count	17 (11, 27)	13 (9, 20)	12 (8, 17)	15 (9, 26)
Unknown	0	0	1	0
Parasite count	57,680 (4446, 254,950)	89,600 (7560, 376,200)	88,000 (8688, 358,200)	68,040 (8987, 264,000)
Sex				
F	88 (48%)	333 (51%)	188 (49%)	75 (44%)
M	94 (52%)	324 (49%)	199 (51%)	94 (56%)

n (%); Median (IQR)

Other was any other form of severe malaria that was not CM or SMA

No associations between the *HMOX1* STR length and any clinical variable directly were identified (Additional file 1: Table S4). In analyses testing for an interaction between STR length and each clinical variable no evidence for differences in STR association by clinical variable were identified (all p for interaction  > 0.05).

Table 4 summarizes the clinical variables available across each malaria subtype.

All analyses were repeated without the addition of PCA’s and clinical sex as covariates. Results were similar, with no major changes in effect estimates.

## Discussion

In the large, multi-centre, MalariaGEN cohort the *HMOX1* STR polymorphism was imputed using a recently published reference panel for STRs. STR imputation could not be tested directly on this cohort, but external validation in other cohorts of similar ancestry showed reasonable imputation accuracy [11]. Across the whole cohort, there was no strong evidence that the length of the *HMOX1* STR altered the risk of severe malaria, or any severe malaria subtypes, although the data was consistent with a small negative effect at some sites, particularly Kenya, Vietnam, and Nigeria. Additionally there was no association with any clinical variables in the cohort for which clinical metadata was available, nor any interaction with clinical variables and outcomes.

The relevance of the *HMOX1* polymorphism in malaria has been recently reviewed [14]. Seven studies have focussed on this question, although all were small and single centre, with a maximum size of 749 patients [15–21]. In all previous studies, STR length was measured using the STR length fragment polymorphism. Nearly all studies used different definitions of STR length (dividing alleles arbitrarily into “short”, “medium” or “long”). Five studies compared development of severe malaria subtypes within cases, with three studies identifying an association with development of severe malaria in cases (strongest association: OR of 0.16 for severe respiratory distress; 95% CI 0.05–0.46 with carriage of a longer allele in Walther et al. [17]), while two studies did not identify any association with development of any severe malaria subtypes. For comparisons of cases and controls, the data were less clear, with only one study (Mendonca et al.) [16], identifying an association between symptomatic malaria and STR length, although this study was largely (90% of cases) in patients with *Plasmodium vivax,* not *Plasmodium falciparum* malaria.

Additionally, a recent large scale phenome wide association study using the *HMOX1* STR across a wide variety of clinical and laboratory parameters was performed in UK Biobank, a large, UK wide cohort study. In this study, there were no robust associations of the *HMOX1* STR repeat [11].

Given the inconsistency of *in-vitro* evidence supporting the biological plausibility of effect, and the suggestion of a differential effect at certain sites (p = 0.06 for heterogeneity), it is worth exploring why the effect might differ at differing sites. Firstly, and most simply, the demographics and recruitment of cases at each site were quite different, with a median age of 29 years in Vietnam, to 1.7 years in Tanzania. Given the strong interactions between severe malaria and age, alongside the myriad of other demographic factors, the impact of the *HMOX1* STR may only occur in certain clinical settings. Secondly, the genetic background at each site was markedly different. As STR’s also represent recent evolution, and the imputation relates to SNP haplotype, it may be that in certain sites these SNPs associate with severe disease, but do not at other sites. Against that, there has been little previous evidence that SNPs in or near *HMOX1* have previously associated with severe malaria [9]. As discussed above, population structure may have also impacted the results, although there was no evidence that estimates varied with or without the use of PCs.

Finally, it may simply represent the play of chance or the microsatellite imparts no functional role in HO-1 expression and/or translation. Given the uncertain previous literature on malaria and the variable functional importance of the repeat in gene expression, together with the weak evidence here, it is possible that there simply is a minimal effect, but that study and population structure influence the results, meaning it is hard to determine conclusively if the effect is present.

## Limitations

The major limitation of work undertaken here is a reliance on imputation, rather than by direct genotyping of the *HMOX1* STR. However, the reliability of this imputation in four separate datasets has been confirmed in recent work [11]; while other published data supports the reliability of the imputation [8, 11]. Secondly, although the imputed STR length was highly correlated with true STR length, it was much less reliable at calling the exact allele length (~ 45% correct), although ~ 80% were called to within 2 repeats, in the validation data. This is partly due to a large number of potential alleles (64 potential STR lengths), with a smaller number of common alleles, making imputation technically challenging [2]. Although this technical limitation should be recognized, it is important to note that all prior associations with this STR (both in and outside malaria) have been with STR length, with no data suggesting the effect is related to a particular allele rather than the total length of the STR [2–5, 14, 17, 22–24]. Additionally, a genome-wide analysis of 2060 expression short tandem STRs found linear associations to be the most common association between STR’s and gene expression, a finding also identified in other studies of human STRs [25–28]. Also, other methods of genotyping STR’s, including fragment length polymorphism measurements used in all previous studies on malaria are also associated with some error in true repeat length due to PCR strand slippage.

Secondly, although principle componsents were used to control for relatedness and population structure within each site, it is well established that PC’s do not completely control for population structure [29]. As ethnic groups are likely to differ in both repeat length and incidence of severe malaria, it may be that population structure within each country is not adequately controlled for in all sites, and the effects identified relate to population stratification. Against that, there were no changes in estimates when using models with and without PC’s, suggesting population stratification did not alter our results.

## Implications

This study is suggestive that *HMOX1* STR variation may not affect the risk of severe malaria, but it remains uncertain whether there may be a suggestive effect, only present under certain environmental conditions. Further research mechanistically linking *HMOX1* genetic variation, gene expression of *HMOX1* and downstream effects on the host–pathogen interaction would be helpful in understanding the role of heme metabolism in severe malaria. Given the potential to therapeutically modulate HMOX1 expression, this might provide a promising avenue for therapeutics in the future [30].

In this large, multisite, study on the *HMOX1* STR polymorphism, including a diverse worldwide population with robust outcome data, results were most consistent with a null effect at most sites, although there was weak indication that STR length might have a small effect in some sites.

## Conclusions

The *HMOX1* promoter STR was not robustly associated with severe malaria, in a large, multi-country cohort.

## Supplementary Information

**Additional file 1: Table S1.** Influence of ethnicity on case status and severe malaria subtypes. **Table S2.** Effect estimates for severe malaria subtypes (values represent OR, 95% CI, and p value). **Table S3.** Effect estimates for the primary outcome using an allelic and genotypic model. **Figure S1.** Effect estimates from the allelic model for all outcomes. **Figure S2.** Effect estimates from the genotypic model. **Table S2.** Differing ethnicities and case status in the Kenya population. **Table S3.** Estimates for the association between HMOX1 repeat length and outcome in a genotypic and alleleic model. **Table S4**. Estimates for the association between HMOX1 repeat length and clinical variables.

## Data Availability

This analysis was performed using the MalariaGEN dataset. Access to this can be arranged via the MalariaGEN website and independent data access committee.
