# Supplementary material for: HMOX1 STR polymorphism and malaria: an analysis of a large clinical dataset
Source: Malar J. 2022 Nov 17;21:342. doi: 10.1186/s12936-022-04352-x (PMC9670449; doi:10.1186/s12936-022-04352-x)
Supplement: Supplementary file 1 — Additional file 1: Table S1. Influence of ethnicity on case status and severe malaria subtypes. Table S2. Effect estimates for severe malaria subtypes (values represent OR, 95% CI, and p value). Table S3. Effect estimates for the primary outcome using an allelic and genotypic model. Figure S1. Effect estimates from the allelic model for all outcomes. Figure S2. Effect estimates from the genotypic model. Table S2. Differing ethnicities and case status in the Kenya population. Table S3. Estimates for the association between HMOX1 repeat length and outcome in a genotypic and alleleic model. Table S4. Estimates for the association between HMOX1 repeat length and clinical variables. [file 12936_2022_4352_MOESM1_ESM.docx]

**Table S1**: Influence of ethnicity on case status and severe malaria subtypes

| Ethnicity | Total number | Cases  ( % total) | SMA  (% cases) | CM  (% cases) | Both  (% cases) |
| --- | --- | --- | --- | --- | --- |
| BurkinaFaso_MOSSI | 1327 | 733 (55.2%) | 28 (3.8%) | 94 (12.8%) | 18 (2.5%) |
| Cameroon_BANTU | 507 | 206 (40.6%) | 26 (12.6%) | 12 (5.8%) | 3 (1.5%) |
| Cameroon_OTHER | 105 | 71 (67.6%) | 9 (12.7%) | 3 (4.2%) | 0 (0%) |
| Cameroon_SEMI_BANTU | 581 | 259 (44.6%) | 31 (12%) | 17 (6.6%) | 5 (1.9%) |
| Gambia_FULA | 857 | 300 (35%) | 66 (22%) | 78 (26%) | 15 (5%) |
| Gambia_JOLA | 686 | 378 (55.1%) | 56 (14.8%) | 123 (32.5%) | 20 (5.3%) |
| Gambia_MANDINKA | 1554 | 791 (50.9%) | 154 (19.5%) | 255 (32.2%) | 45 (5.7%) |
| Gambia_OTHER | 1222 | 705 (57.7%) | 118 (16.7%) | 233 (33%) | 39 (5.5%) |
| Gambia_WOLLOF | 720 | 305 (42.4%) | 61 (20%) | 90 (29.5%) | 14 (4.6%) |
| Ghana-Kumasi_AKANS[ASHANTI_EASTERN] | 158 | 92 (58.2%) | 18 (19.6%) | 13 (14.1%) | 1 (1.1%) |
| Ghana-Kumasi_NORTHERNER | 11 | 11 (100%) | 5 (45.5%) | 1 (9.1%) | 0 (0%) |
| Ghana-Kumasi_OTHER | 55 | 38 (69.1%) | 1 (2.6%) | 1 (2.6%) | 0 (0%) |
| Ghana-Noguchi_KASEM | 264 | 130 (49.2%) | 10 (7.7%) | 5 (3.8%) | 3 (2.3%) |
| Ghana-Noguchi_NANKAM | 183 | 90 (49.2%) | 3 (3.3%) | 8 (8.9%) | 0 (0%) |
| Ghana-Noguchi_OTHER | 45 | 35 (77.8%) | 4 (11.4%) | 3 (8.6%) | 1 (2.9%) |
| Kenya_CHONYI | 1010 | 369 (36.5%) | 49 (13.3%) | 150 (40.7%) | 41 (11.1%) |
| Kenya_GIRIAMA | 1648 | 945 (57.3%) | 106 (11.2%) | 409 (43.3%) | 110 (11.6%) |
| Kenya_KAUMA | 308 | 129 (41.9%) | 10 (7.8%) | 55 (42.6%) | 13 (10.1%) |
| Kenya_OTHER | 249 | 159 (63.9%) | 9 (5.7%) | 74 (46.5%) | 25 (15.7%) |
| Malawi_MALAWI | 2499 | 1182 (47.3%) | 65 (5.5%) | 642 (54.3%) | 109 (9.2%) |
| Mali_BAMBARA | 203 | 114 (56.2%) | 34 (29.8%) | 18 (15.8%) | 28 (24.6%) |
| Mali_BAMBARA_MIXED | 32 | 26 (81.2%) | 6 (23.1%) | 10 (38.5%) | 7 (26.9%) |
| Mali_MALINKE | 44 | 29 (65.9%) | 8 (27.6%) | 7 (24.1%) | 6 (20.7%) |
| Mali_OTHER | 119 | 69 (58%) | 21 (30.4%) | 20 (29%) | 10 (14.5%) |
| Mali_PEULH | 24 | 11 (45.8%) | 6 (54.5%) | 3 (27.3%) | 0 (0%) |
| Mali_SARAKOLE | 24 | 14 (58.3%) | 6 (42.9%) | 3 (21.4%) | 0 (0%) |
| Nigeria_OTHER | 8 | 7 (87.5%) | 0 (0%) | 1 (14.3%) | 0 (0%) |
| Nigeria_YORUBA | 123 | 102 (82.9%) | 1 (1%) | 27 (26.5%) | 0 (0%) |
| PapuaNewGuinea_MADANG | 485 | 275 (56.7%) | 88 (32%) | 34 (12.4%) | 3 (1.1%) |
| PapuaNewGuinea_MADANG_MIXED | 27 | 16 (59.3%) | 2 (12.5%) | 0 (0%) | 0 (0%) |
| PapuaNewGuinea_OTHER | 87 | 48 (55.2%) | 14 (29.2%) | 7 (14.6%) | 2 (4.2%) |
| PapuaNewGuinea_SEPIK | 67 | 37 (55.2%) | 9 (24.3%) | 7 (18.9%) | 2 (5.4%) |
| Tanzania_MZIGUA | 200 | 104 (52%) | 51 (49%) | 9 (8.7%) | 13 (12.5%) |
| Tanzania_MZIGUA_MIXED | 51 | 28 (54.9%) | 15 (53.6%) | 0 (0%) | 1 (3.6%) |
| Tanzania_OTHER | 150 | 74 (49.3%) | 23 (31.1%) | 7 (9.5%) | 5 (6.8%) |
| Tanzania_WABONDEI | 92 | 45 (48.9%) | 10 (22.2%) | 4 (8.9%) | 1 (2.2%) |
| Tanzania_WABONDEI_MIXED | 61 | 33 (54.1%) | 16 (48.5%) | 2 (6.1%) | 1 (3%) |
| Tanzania_WASAMBAA | 171 | 82 (48%) | 46 (56.1%) | 6 (7.3%) | 3 (3.7%) |
| Tanzania_WASAMBAA_MIXED | 82 | 43 (52.4%) | 17 (39.5%) | 3 (7%) | 1 (2.3%) |
| Vietnam_KINH | 1163 | 628 (54%) | 18 (2.9%) | 146 (23.2%) | 4 (0.6%) |
| Vietnam_OTHER | 33 | 32 (97%) | 1 (3.1%) | 4 (12.5%) | 0 (0%) |
| Vietnam_S_TIENG | 50 | 40 (80%) | 4 (10%) | 2 (5%) | 0 (0%) |
| NA | 304 | 146 (48%) | 3 (2.1%) | 6 (4.1%) | 1 (0.7%) |

**Table S2**: Effect estimates for severe malaria subtypes (values represent OR, 95% CI, and p value)

| country | CM_vs_case | CM_vs_control | SMA_vs_case | status | SMA_vs_control | BOTH_vs_control | BOTH_vs_case |
| --- | --- | --- | --- | --- | --- | --- | --- |
| BurkinaFaso | 1.47 (1.11-1.93),0.0068 | 1.32 (0.97-1.81),0.0783 | 1.08 (0.68-1.74),0.7377 | 1.01 (0.84-1.22),0.9065 | 1.1 (0.66-1.84),0.7193 | NA | NA |
| PNG | 1.11 (0.56-2.2),0.7578 | 1.39 (0.66-2.92),0.3804 | 1.14 (0.68-1.91),0.6162 | 1.32 (0.94-1.85),0.1042 | 1.29 (0.77-2.17),0.3297 | NA | NA |
| Malawi | 0.89 (0.77-1.02),0.102 | 0.95 (0.84-1.07),0.3924 | 1.32 (0.95-1.83),0.0997 | 1 (0.91-1.11),0.9534 | 1.29 (0.93-1.8),0.1275 | 1.22 (0.95-1.57),0.1161 | 1.2 (0.94-1.54),0.1445 |
| Cameroon | 1.01 (0.64-1.59),0.9715 | 1.13 (0.63-2.02),0.6923 | 1.27 (0.9-1.8),0.1784 | 1.05 (0.86-1.28),0.6357 | 1.07 (0.63-1.82),0.813 | NA | NA |
| Mali | 0.88 (0.59-1.32),0.5435 | 0.94 (0.64-1.38),0.7409 | 1 (0.7-1.42),0.995 | 1 (0.78-1.29),0.9797 | 1.01 (0.71-1.43),0.9744 | 1.1 (0.7-1.71),0.6811 | 1.21 (0.79-1.85),0.3893 |
| Ghana | 1.13 (0.72-1.76),0.5881 | 0.92 (0.58-1.46),0.7195 | 1.05 (0.7-1.57),0.8059 | 0.86 (0.71-1.03),0.1017 | 0.87 (0.58-1.31),0.5004 | NA | NA |
| Vietnam | 1.08 (0.77-1.52),0.644 | 0.74 (0.51-1.07),0.1118 | 0.91 (0.36-2.26),0.8316 | 0.75 (0.59-0.95),0.0183 | 0.53 (0.2-1.39),0.1946 | NA | NA |
| Kenya | 1.07 (0.94-1.22),0.297 | 0.93 (0.83-1.06),0.2708 | 1 (0.81-1.24),0.9733 | 0.9 (0.82-0.99),0.0296 | 0.9 (0.73-1.1),0.301 | 0.8 (0.66-0.98),0.0344 | 0.89 (0.72-1.09),0.2469 |
| Gambia | 1.03 (0.92-1.15),0.624 | 1.04 (0.93-1.16),0.5089 | 1.01 (0.88-1.16),0.8791 | 1.02 (0.94-1.1),0.6415 | 1.03 (0.9-1.18),0.6721 | 0.89 (0.7-1.13),0.3492 | 0.85 (0.68-1.08),0.1861 |
| Tanzania | 0.95 (0.58-1.57),0.8487 | 0.83 (0.51-1.36),0.4569 | 0.94 (0.72-1.23),0.6397 | 0.91 (0.76-1.1),0.3445 | 0.9 (0.71-1.14),0.3895 | 0.92 (0.52-1.63),0.7802 | 0.98 (0.56-1.7),0.9393 |
| Nigeria | 0.44 (0.22-0.89),0.0221 | NA | NA | 0.54 (0.28-1.07),0.079 | NA | NA | NA |
| Meta-analysis | 1.02 (0.92-1.13),0.6713 | 0.98 (0.92-1.05),0.5401 | 1.04 (0.95-1.13),0.3654 | 0.96 (0.91-1.02),0.1718 | 1 (0.91-1.09),0.9425 | 0.96 (0.8-1.16),0.6818 | 0.99 (0.84-1.17),0.8899 |

**Table S3:** Effect estimates for the primary outcome using an allelic and genotypic model

| term | Effect estimate | P value |
| --- | --- | --- |
| Genotypic model, ref = MM | | |
| MS | 1.15 (1.01-1.3) | 0.0361 |
| LM | 1.11 (0.99-1.25) | 0.0622 |
| LS | 1.11 (0.96-1.28) | 0.1686 |
| SS | 1.11 (0.87-1.42) | 0.4036 |
| LL | 1.05 (0.88-1.25) | 0.6030 |
| Allelic model, number of containing alleles | | |
| S allele | 1.05 (0.98-1.14) | 0.1813 |
| L allele | 0.98 (0.92-1.05) | 0.5569 |
| M allele | 0.97 (0.91-1.05) | 0.4575 |

**Figure S1: Effect estimates from the allelic model for all outcomes.**

**
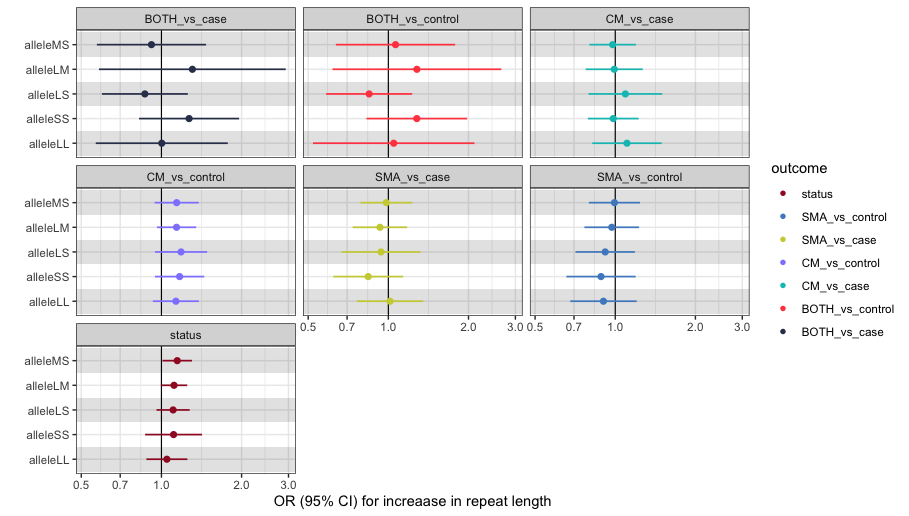
**

**Figure S2: Effect estimates from the genotypic model**

**
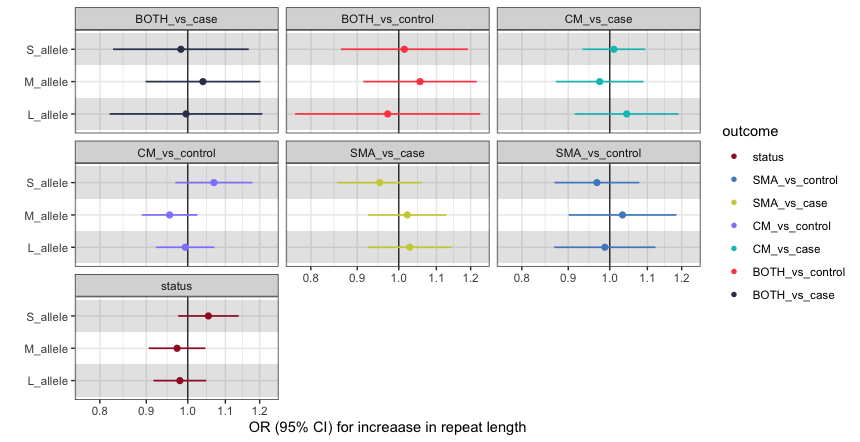
**

**Table S4:** Estimates for the association between HMOX1 repeat length and clinical variables

| name | estimate | p.value |
| --- | --- | --- |
| bpd ~ length | -0.03 (-0.15,0.09) | 0.6554047 |
| bps ~ length | -0.08 (-0.22,0.05) | 0.2180671 |
| platelet ~ length | 0.21 (-0.77,1.19) | 0.6766121 |
| mcv ~ length | 0.05 (-0.02,0.11) | 0.1445391 |
| wbc ~ length | 0.01 (-0.08,0.09) | 0.8770553 |
| parasite_log ~ length | 0 (-0.01,0.02) | 0.5761085 |
